# Setting goals for pollinator gardens

**DOI:** 10.1111/cobi.70009

**Published:** 2025-03-12

**Authors:** Nicholas N. Dorian, Atticus W. Murphy, Amy M. Iler, Paul J. CaraDonna

**Affiliations:** ^1^ Program in Plant Biology & Conservation Northwestern University Evanston Illinois USA; ^2^ Negaunee Institute for Plant Conservation Science and Action Chicago Botanic Garden Glencoe Illinois USA; ^3^ Department of Biology Tufts University Medford Massachusetts USA; ^4^ Department of Evolution & Ecology University of California, Davis Davis California USA

**Keywords:** adaptive management, biodiversity conservation, community‐based conservation, goal setting, insect, science communication, urban gardens, yard management, comunicación de la ciencia, conservación basada en la comunidad, conservación de la biodiversidad, establecimiento de objetivos, insectos, jardines urbanos, manejo adaptativo, manejo de jardines, 生物多样性保护, 目标设定, 适应性管理, 昆虫, 基于社区的保护, 庭院管理, 科学交流, 城市花园

## Abstract

In recent years, declines in animal pollinators have stimulated tremendous interest in pollinator‐friendly gardening. There is a widespread notion that pollinator gardens are beneficial, but the specific capacity of pollinator gardens to improve biodiversity conservation and societal well‐being remains unclear. We argue that setting clear ecological and social goals can clarify the value of pollinator gardens for both pollinators and people. Effective goals will articulate specific, quantifiable, and realistic endpoints across scales of biological organization. Opportunities and challenges for setting goals will vary across landscape contexts, cultural systems, stakeholder values, and geographic regions. In community‐based pollinator projects, harnessing the potential of gardens to improve outcomes requires an evidence‐based, iterative process involving identifying shared values, defining specific goals and measurable indicators, proposing straightforward interventions, monitoring progress, and evaluating success, including adaptive management if success is not met. These ideas provide ecologists and conservation practitioners with a practical framework for how to channel the swell of enthusiasm for pollinator gardening and, more generally, community‐driven conservation efforts in dynamic socioecological systems toward measurable impacts on biodiversity and people.

## Abstract (es)

Objetivos para los jardines de polinizadores

**Resumen:**

Recientemente, el declive de los animales polinizadores ha despertado un enorme interés por la jardinería amigable con los polinizadores. Existe la idea generalizada de que los jardines de polinizadores son beneficiosos, pero sigue sin estar clara la capacidad específica de estos jardines para mejorar la conservación de la biodiversidad y el bienestar de la sociedad. Sostenemos que el establecimiento de objetivos ecológicos y sociales claros puede aclarar el valor de los jardines de polinizadores tanto para los polinizadores como para las personas. Los objetivos efectivos articularán puntos finales específicos, cuantificables y realistas en todas las escalas de organización biológica. Las oportunidades y los retos a la hora de establecer objetivos variarán en función de los contextos paisajísticos, los sistemas culturales, los valores de las partes interesadas y las regiones geográficas. En los proyectos comunitarios sobre polinizadores, aprovechar el potencial de los huertos para mejorar los resultados requiere un proceso iterativo basado en pruebas que incluya la identificación de valores compartidos, la definición de objetivos específicos e indicadores mensurables, la propuesta de intervenciones sencillas, el seguimiento de los progresos y la evaluación del éxito, incluida la gestión adaptativa si no se alcanza el éxito. Estas ideas proporcionan a los ecólogos y a los profesionales de la conservación un marco práctico para canalizar el entusiasmo por la jardinería de polinizadores y, más en general, los esfuerzos de conservación impulsados por la comunidad en sistemas socioecológicos dinámicos, hacia impactos medibles sobre la biodiversidad y las personas.

## 为传粉者花园设定目标

【摘要】 近年来, 动物传粉者的丧失激发了人们对传粉者友好型园艺的极大兴趣。人们普遍认为传粉者花园是有益的, 但其在促进生物多样性保护和提升社会福祉方面的具体能力仍不清楚。我们认为, 制定清晰的生态和社会目标可以明确传粉者花园对传粉者和人类的价值。有效的目标将阐明不同尺度的生物组织具体、可量化和现实的终点。在不同的景观背景、文化体系、利益相关者价值观和地理区域之间, 制定目标的机遇和挑战也各不相同。在基于社区的传粉者项目中, 利用花园的潜力来改进保护成效需要一个基于证据的迭代过程, 包括确定共同价值观、定义具体目标和可衡量指标、提出直接干预措施、监控进展, 以及评估成功与否, 这也包括在未取得成功时进行适应性管理。这些想法为生态学家和保护工作者提供了一个实用的框架, 帮助他们了解如何在动态的社会生态系统中引导人们对传粉者园艺的热情, 以及更普遍地推进基于社区的保护工作, 从而对生物多样性和人类产生可衡量的影响。**【翻译:胡怡思;审校:聂永刚】**

## INTRODUCTION

Over the past 2 decades, there has been a growing movement to conserve animal pollinators. Pollinators, such as bees, butterflies, and hummingbirds, provide critical pollination services to wild plants and agricultural crops (Klein et al., 2007), and their observed declines (Potts et al., 2010; Wagner, 2020) have prompted an all‐hands‐on‐deck approach to identifying best practices for rescuing pollinator populations and their associated ecosystem services (Baldock, 2020; Senapathi et al., 2015). One practice that has gained popularity is the planting of pollinator gardens, which are areas that provide flowers, nesting sites, and host plants for animal pollinators without pesticides (Majewska & Altizer, 2020; Shepherd et al., 2003). To date, more than 1 million pollinator gardens have been planted in the United States, and more than 45,000 gardens have been registered with the program Monarch Watch specifically to help monarch butterflies (National Pollinator Garden Network, 2019; Taylor, 2017).

One reason pollinator gardens are popular is the notion that they are well positioned to benefit both pollinators and people. Gardens are thought to have high potential for pollinator conservation because they appear to provide food and nesting resources for a diverse community of flower‐visiting insects and because a large proportion of residential lands currently maintained as turf grass could potentially be converted to flower‐rich pollinator habitat (Lerman et al., 2023; Majewska & Altizer, 2020). Similarly, gardens are thought to benefit people because gardens can increase environmental regulation, recreational opportunities, mental and physical well‐being, and human–nature connection (Baldock, 2020; Bateman, 2023; Haase & Gaeva, 2023).

**FIGURE 1:**
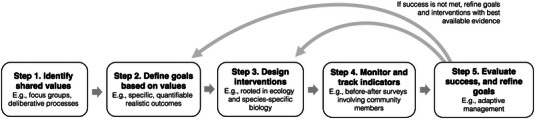
Steps to incorporate goals in conservation planning for pollinator gardens.

**FIGURE 2:**
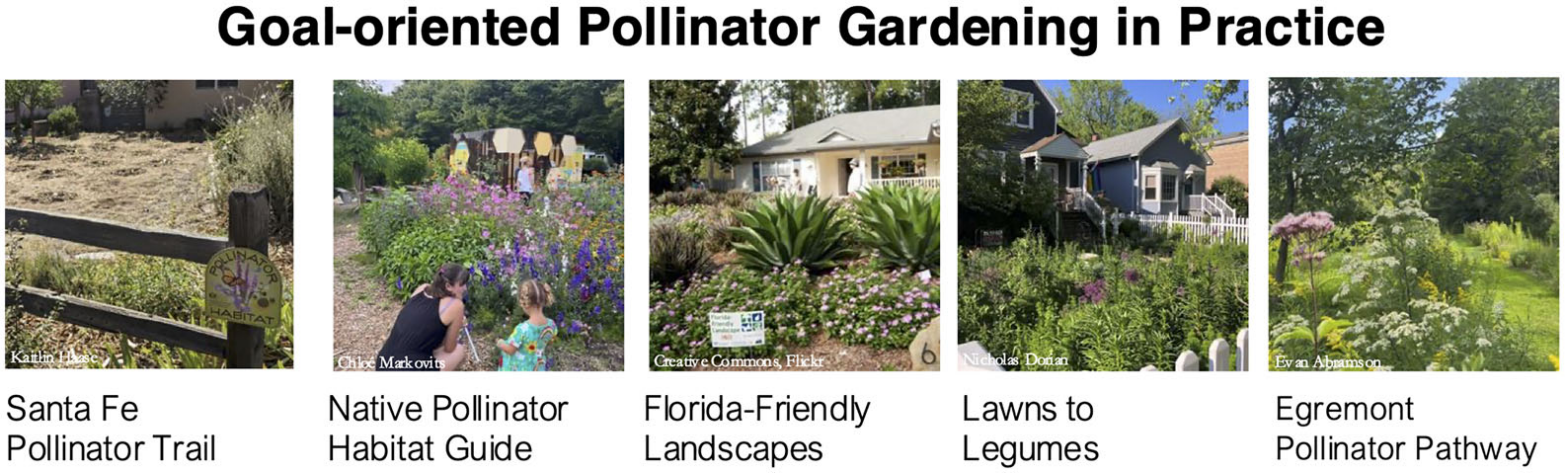
Goal‐oriented pollinator gardening projects from the United States. See Table 2 for details and references on each project.

We were inspired to write this essay because we noticed a potential mismatch between enthusiasm for what pollinator gardens might be able to achieve and knowledge of what they actually can and cannot achieve. We argue that understanding the value of pollinator gardens requires setting clear goals. When specific, measurable goals are defined up front, it becomes possible to know whether or not pollinator gardens are working. For instance, pollinator gardens often attract impressive numbers of flower‐visiting insects (Majewska & Altizer, 2020), but is this indicative of conservation success? How success is defined (or not defined) makes all the difference in answering this question. Integrating goals into current practice can deepen understanding of whether gardens can achieve ecological goals that aim to benefit pollinators across scales of biological organization and social goals that aim to benefit people. Key to harnessing the potential of goals is embedding goal setting in an evidence‐based, iterative process (Figure 1). When effective goals anchor the design of interventions and interventions are evaluated across space and time, it becomes possible to clarify the value of pollinator gardens. We considered goal‐setting opportunities for pollinator gardens through the lens of community‐based projects because these are the most common circumstances in which scientists have the opportunity to collaborate on a goal‐setting framework (Figure 2; Table 2). We hope this essay inspires diverse practitioners of pollinator gardening to embrace the idea that articulating goals is a key step to clarifying the impact of their actions and helping their gardens meet their conservation potential.

## SETTING GOALS

Clear goals chart the path forward. Setting goals prompts practitioners to specify aims rooted in values (i.e., What endpoint is desired?), to guide the plan for action (i.e., What interventions will help achieve goals?), and to decide on how best to monitor progress toward achieving those goals (i.e., What needs to be measured to evaluate success?) (Miller & Hobbs, 2007). Although goal setting is not new to pollinator conservation (Menz et al., 2011), we think it could be made more explicit in the current practice of pollinator gardening. To investigate this perception, we described the goals of 43 pollinator initiatives in Massachusetts, United States, a region with a strong grassroots movement around pollinator gardening and where 2 of us interfaced with community groups on pollinator gardening over 5 years (Appendix S1). We aimed to characterize the types of goals being set by a particularly well‐documented group of pollinator gardeners at this moment in time. We identified 52 goals, the majority of which dealt with ecological themes (41 of 52). Although these ecological goals were oriented toward pollinator conservation, many of them were not specific and hard to measure (e.g., support pollinators or promote biodiversity, 34 of 41).

Effective goals for pollinator gardens will be specific, quantifiable, and realistic (Beier et al., 2016; Butchart et al., 2016; Miller & Hobbs, 2007; Table 1). By *specific*, we mean goals focused on a particular aspect of biodiversity whether at the level of the population (e.g., recovery of at‐risk taxa); the community (e.g., restoration of functional trait diversity or species interactions); or the ecosystem (e.g., restoration of pollination services). By *quantifiable*, we mean developing goals around performance criteria or indicators that can be measured. This could mean, for example, at the population level, a target number of individuals of the focal taxa is reached; at the community level, a target structural property of a plant–pollinator network is achieved; or at the ecosystem level, a target fruit set is attained in crops growing adjacent to gardens (Butchart et al., 2016; Prach et al., 2019) (Table 1). And, by *realistic*, we mean setting goals that can actually be achieved. This means aiming goals at species and communities that occur where gardens are planted or focusing on ecosystem processes that occur at the scale of the garden or multiple gardens (Ehrenfeld, 2000; Goddard et al., 2010) (Table 1). A corollary to this is that some goals may not be achievable, such as restoring taxa that have minimum area requirements that exceed the scale of a garden or taxa that have stringent habitat needs that cannot be provided in a garden (Goddard et al., 2010). In addition, what is realistic for one region may be completely unrealistic for another region because of variation in climate, landscape context, economic barriers (e.g., funding), or social constraints (e.g., public acceptance) (Miller & Hobbs, 2007). Ensuring that goals are realistic is particularly important for retaining long‐term interest and participation from community practitioners.

**TABLE 1:** Examples of specific, quantifiable, and realistic goals that can be used to clarify the conservation value of pollinator gardens.

Goal type	Scale	Specific goal	Quantifiable target	Proposed intervention	Emergent research question
Ecological	Population	Support at‐risk taxa and/or flagship taxa^a^	Increase abundance of target taxa in gardens	Design gardens around specific habitat requirements to support focal species	Is increased occupancy in gardens indicative of demographic increases?
Ecological	Community (biodiversity)	Support pollinators sensitive to land use change, e.g., diet specialists^b^	Presence of species with target functional traits in plantings	Design gardens using local interaction data to attract species with target functional trait	How does restoration influence community assembly after landscape simplification?
Ecological	Community (species interactions)	Restore resilient plant–pollinator visitation network^c^	Increase in network metrics of resilience, e.g., connectance compared to ornamental gardens	Design gardens to be attractive to diverse insect pollinators	How does plant–pollinator network structure vary across growing seasons (time) and gardens (space)?
Ecological	Ecosystem	Restore pollination deficits to urban crops^d^	Increase fruit set of target crops	Design gardens with plants known to attract crop pollinators	How does spatial arrangement and scale of gardens influence delivery of pollination services?
Social	Individual person/neighborhood of people	Strengthen human–nature connectedness^e^	Increase in nature‐connectedness ranking according to standardized scales	Design gardens to attract conspicuous insects to help residents notice nature	How can environmental education modulate feelings of nature connectedness in gardeners?

*Note*: These goals are inspired by studies from the broader conservation literature and our own work engaging with the public on pollinator gardening. They showcase the broad diversity of goals that could be set for pollinator gardening and in doing so stimulate conversation about specific, measurable endpoints for pollinator gardens.

^a^ Abramson (2021b).

^b^ Kremen & M'Gonigle (2015).

^c^ Forup et al. (2008).

^d^ Zink et al. (2023).

^e^ Barragan‐Jason et al. (2022).

Specific, quantifiable, and realistic goals were present in our sample of pollinator gardening initiatives. About one fifth of ecological goals (8 of 41) pertained to restoration of regionally at‐risk pollinators. For example, one initiative focused on increasing bumble bee (*Bombus* spp.) species richness by installing a demonstration native plant garden in a public park. The project team compared species composition at the site before and after planting and quantified success in 2 ways: an increase in overall bumble bee diversity and an increase in the occupancy of 3 at‐risk bumble bee species in the northeast (Abramson, 2021b). This project exemplifies the sort of goal setting for which we advocate. Their goals were specific because they focused on a particular group of pollinators, quantifiable because success had measurable outcomes of diversity and occupancy, and realistic because goals were informed by local knowledge of species that could be targeted by gardens in their area.

Given that biodiversity conservation on residential lands is aincreasingly becoming an important component of people's cultural experience (Lerman et al., 2023), pollinator gardening presents a ripe opportunity to set social goals in tandem with ecological goals (Standish et al., 2013). In our sample of pollinator gardening initiatives from Massachusetts, about one fifth of all goals (11 of 52) dealt with social themes, mostly having to do with environmental education. Social goals are centered on achieving benefits to people through the practice of gardening, such as strengthening human–nature connectedness, improving ecological literacy, creating aesthetic landscapes, building neighborhood ties, or enhancing physical well‐being (Baldock, 2020; Bateman, 2023; Haase & Gaeva, 2023; Wells et al., 2023; Table 1).

Variation in land‐use history, climate, demographics, and pollinator communities generates diverse opportunities and challenges for setting goals. To demonstrate the outcomes of this variation, we highlight 5 goal‐oriented pollinator gardening projects from across the United States (Figure 2; Table 2). These examples are united in that they all try to achieve benefits for people or pollinators through gardens, but they vary in their stated goals and actions to accomplish those goals. Each project faces unique barriers to success, which stem from the environmental or social context in which the project occurs. We chose these examples to demonstrate the breadth of contexts in which pollinator gardens occur in the United States and acknowledge that they may not fully represent the opportunities and challenges of setting goals in other socioecological contexts worldwide. Still, we contend that our goal‐setting framework remains relevant regardless of region for understanding what additions of flower‐rich areas on residential lands can and cannot achieve for pollinators and people.

**TABLE 2:** Goal‐oriented pollinator gardening projects from the United States to showcase diverse opportunities and challenges for setting goals.

	Project				
	Santa Fe Pollinator Trail^a^	Native Pollinator Habitat Guide^b^	Florida‐Friendly Landscapes^c^	Lawns to Legumes^d^	Egremont Pollinator Pathway^e^
Region	Southwest (New Mexico)	Northwest (Washington)	Southeast (Florida)	Midwest (Minnesota)	Northeast (Massachusetts)
Context	Urban	Urban or suburban	Suburban	Suburban	Rural
Goals	Restore resources for pollinators, especially native bees Create pathway of habitats across city	Educate public Excite public through garden aesthetics	Reduce stormwater runoff Create pollinator habitat in community landscapes	Create pollinator habitat in yards Build public acceptance for nonconventional yards	Conserve at‐risk bumble bees and Lepidoptera Raise awareness for pollinators
Interventions	Distribute garden kits to city residents Vary garden kits across contexts, e.g., backyard, school	Facilitate learning with signage, trails Involve long‐term stewards, especially community, rural, tribal leaders	Replace turf grass with flower‐rich gardens Engineer gardens to capture rainwater	Provide garden options, e.g., balcony, bee lawn Offer online and in‐person educational resources	Link garden plants to needs of at‐risk pollinators Engage volunteers in demonstration garden planting
Measuring success	No. of plants installed No. of new gardens planted across city	n/a	No. of registered yards with Florida‐Friendly program	No. gardens planted No. of people trained	Increase in bumble bee diversity, incl. targets *Bombus vagans*, *Bombus fervidus*, *Bombus terricola*
Barriers to success	Prolonged drought hampers plant establishment High impervious surface limits garden locations	Mismatched aesthetic norms and pollinator needs Sightlines require low‐growing plants	Home owner association landscaping restrictions Desire for social capital discourages lawn conversion	Stinging insects discourage participation Difficult to establish forbs in lawn	Invasive plants threaten long‐term garden viability

^a^ Haase (2023).

^b^ Schwartz and Salisbury (2018).

^c^ UF/IFAS Extension (2024).

^d^ Shaw et al. (2020).

^e^ Abramson (2021a).

## GOAL‐ORIENTED PROCESS

To clarify the value of pollinator gardens, goal setting must be embedded in an iterative process (Figure 1). We devised a process inspired by conservation biology (Tear et al., 2005), ecological restoration (Miller & Hobbs, 2007), and community‐based conservation (Kirk et al., 2021; Turo & Gardiner, 2020) that emphasizes the importance of working with project stakeholders to coproduce goals that guide the development of management interventions, monitoring protocols, and project evaluation (Beier et al., 2016).

### Identify shared values (Step 1)

In community‐based projects, finding common ground is key to articulating goals that will be agreed upon by all. Stakeholders will have values shaped by diverse knowledge systems, philosophies, and social institutions that may initially lead to divergent opinions of ideal goals for gardens (Turo & Gardiner, 2020). Democratically including diverse perspectives into goal setting requires a deliberate, group‐based process that translates intrinsic values of stakeholders into specific contextual values (Ranger et al., 2016). Shared values can be identified by actively soliciting input from diverse community members and nontraditional participants through focus groups, neighborhood meetings, or online surveys.

### Define goals that reflect shared values (Step 2)

Cocreating goals with community members means building goals that explicitly show how values were incorporated and why endpoints have local relevance (Beier et al., 2016). One approach is “backwards design,” which lets desired results (e.g., What does success look like?) and project parameters (e.g., What is the project budget and timeline?) guide goal development (Varner, 2014). Ensuring that values of all stakeholders are represented explicitly in objectives from the project outset is key for lasting community involvement (Parker, 2010; Phalen, 2009). Disagreement among stakeholders over project goals and priorities may arise, so it is important to take time up front to find consensus to ensure project support in the long term (Beier et al., 2016).

Often, multiple objectives will emerge from goal‐setting discussions (Kirk et al., 2021). Ecological goals need not be siloed from social goals, as long as they are accompanied by careful consideration of how the 2 types of goals interact. In some cases, ecological and social goals interact in synergy. For example, social aims to mitigate water use through xeriscaping can simultaneously advance ecological aims to support biodiversity by motivating pollinator gardening with drought‐tolerant native plants. In other cases, dissonance may arise between ecological and social goals. For example, providing complex habitat structure for nesting pollinators may conflict with a desire for a manicured landscape. In this example, to reconcile incompatible goals, one solution could be landscaping with “cues to care,” which are management actions that indicate intentional human intervention (e.g., yard signs, edged beds) (Nassauer, 1995). Delineating goals that are rooted in stakeholder values can lead to more nuanced interpretations of success and a more holistic understanding of what conservation looks like in dynamic socioecological systems.

### Design interventions based on goals (Step 3)

Interventions to achieve specific goals will necessarily be rooted in ecology and species‐specific biology of the target taxa, community of taxa, or ecosystem process (Glenny et al., 2023; Kremen & M'Gonigle, 2015; Menz et al., 2011). Interventions will also be developed around structural elements designed for the scale of the garden and of the landscape in which the garden is being installed. For example, a garden designed to increase the reproductive output of monarch butterflies (*Danaus plexippus*) might focus on the host plant common milkweed (*Asclepias syriaca*) and suitable nectar plants for adults during breeding (summer) and migration (fall), and garden designs could be developed that are suitable for a range of contexts (e.g., urban balcony vs. suburban yard). Offering multiple evidence‐based interventions to community members can provide a sense of agency and ownership over the look of their garden while still ensuring that installed gardens are contributing to a particular goal. We call this an IKEA‐style approach in that garden designs are not only evidence based but also simple, practical, and affordable for community members to implement and maintain. Multiple options can lead to a mosaic of goal‐oriented gardens across the landscape such that no single garden type or plant palette dominates.

### Monitor progress of interventions toward achieving goals (Step 4)

Methods for monitoring progress should be accurate, feasible, consistently employed, and cost‐effective (Tear et al., 2005). For ecological goals, we advocate for monitoring taxa or ecological processes that can be easily identified or tracked (e.g., a nonlethal morphogroup approach or field‐identifiable taxa [Dorian et al., 2024; Erickson et al., 2022]) and for implementing monitoring using a replicated before–after design to gauge impact of a particular intervention. Both short‐ and long‐term monitoring of quantifiable indicators will be important for assessing outcomes (Prach et al., 2019; Tear et al., 2005), especially because pollinator populations can be highly variable across space and time (Aldercotte et al., 2022; Ogilvie & CaraDonna, 2022; Vázquez et al., 2023). For social goals, informal surveys could be delivered to attendees of an event to gauge baseline understanding or environmental awareness, or surveys delivered at the beginning and end of a course could be used to gauge measurable changes in ecological literacy or connectedness to nature in students (Varner, 2014). Creating ways for community members to measure the impact of their actions directly through monitoring can work toward social objectives by fostering feelings of agency, developing a stronger sense of place, fostering an ethic of care and respect for insects, and building lasting people–pollinator relationships (Beckwith et al., 2022; Knapp et al., 2021; Samways et al., 2020). Involving the public in data collection also means shifting who produces and shares ecological knowledge, from a single top‐down direction (scientist to gardener) to a bidirectional flow between scientists and gardeners.

### Evaluate success and refine goals if success is not met (Step 5)

Conservation in changing landscapes is a moving target, not the least because social and ecological systems are inherently dynamic. In practice, evaluation of management interventions may reveal that goals were not achieved as initially planned. Barriers to success include ecological factors (e.g., plant establishment, restricted patch size, invasive species), social factors (e.g., public acceptance, aesthetic norms, insufficient representation of all stakeholders), and economic factors (e.g., financial constraints; Table 2). Identifying what did and did not work can generate fruitful areas for future research. For ecological goals, much still remains to be learned about basic pollinator biology, including nutritional needs, nesting requirements, and movement, as well as ecological processes, including source–sink dynamics of pollinators in gardens and the importance of spatial scale for provisioning ecosystem services (Dorian et al., 2024; Goddard et al., 2010; Lepczyk et al., 2017). Similarly, research investigating the feedback between human decision‐making and ecological dynamics is needed to gain a fuller understanding of conservation in human‐altered environments (Chinga et al., 2024; Martin et al., 2024). These knowledge gaps can be embraced through adaptive management, which is a process that seeks to improve the effectiveness of conservation management by updating goals and interventions with the best available evidence (Gillson et al., 2019) (returning to steps 2 and 3 in Figure 1).

Putting it all together, a community‐based collaboration toward effective pollinator gardening can lead to a public document containing a summary of findings from focus groups and listening sessions, including values of community members; articulation of specific project goals, objectives and indicators of success; a concise catalog of interventions (e.g., garden designs) to achieve specific goals in a variety of contexts; a timeline and protocol for community‐engaged monitoring of indicators and outreach; and plans for reevaluating project success following monitoring. Communicating findings to relevant stakeholders in a way that is readily understood ensures transparency and can help retain public interest in the project over the long term.

## CLOSING THOUGHTS

Over the past 2 decades, gardens on residential and public lands have emerged as an exciting frontier in pollinator conservation. Community groups have been remarkably effective at self‐organizing, spreading ideas about pollinator‐friendly gardening, and dedicating their time toward making positive impact. We hope our essay helps elevate these efforts by outlining why specific, measurable objectives are needed to realize the full potential of pollinator gardens. Setting clear goals for pollinator gardens is as much a chance to achieve desirable benefits for biodiversity and people as it is an opportunity to learn what gardening on residential lands can and cannot accomplish. Although we dealt with the specific case of pollinator gardening, we think our goal‐setting framework remains relevant for conservation in other socioecological systems, particularly those occurring in urban and suburban settings. It will always be challenging to achieve success in these situations, especially because there is much to understand about the ecology of human‐managed systems. However, when time is taken to explicate concrete goals up front and to thoughtfully balance the values of diverse stakeholders, it becomes easier to know where a project is headed and to measure whether it has been a success.

## AUTHOR CONTRIBUTIONS

Nicholas N. Dorian and Paul J. CaraDonna jointly conceived of these ideas, in part through conversations with Atticus W. Murphy and Amy M. Iler. Nicholas N. Dorian wrote the first draft of the manuscript. Nicholas N. Dorian, Atticus W. Murphy, Amy M. Iler, and Paul J. CaraDonna jointly revised the manuscript.

## Supporting information



Supporting information
