# Supplementary material for: Setting goals for pollinator gardens
Source: Conserv Biol. 2025 Mar 12;39(4):e70009. doi: 10.1111/cobi.70009 (PMC12309651; doi:10.1111/cobi.70009)
Supplement: Supplementary file 1 — Supporting information [file COBI-39-e70009-s001.docx]

**Supporting Information for SETTING GOALS FOR POLLINATOR GARDENS**

Last updated: 23 January 2025

**Appendix S1**

Description: Survey of goals set by pollinator gardening initiatives in Massachusetts, USA.

Based on the work of two authors in Massachusetts engaging communities about pollinator gardening, and discussions among all authors, we developed the perception that goal-setting is missing from current practice. To investigate this perception, we characterized the state of community-based pollinator gardening efforts in Massachusetts. In this region, pollinator gardening has grown sharply in popularity over the past five years and is primarily performed by small organizations run by gardeners without training in biodiversity restoration or conservation. In other words, we aimed to capture the recent trends in pollinator gardening and the perspectives of diverse, non-expert conservation practitioners. We acknowledge that trends in Massachusetts may not be similar to those of other regions, however, we use our sample of goal setting in pollinator gardens to motivate our perspective piece rather than to make definitive statements about the approach of all pollinator gardeners.

From September–October 2023, we conducted an internet search for pollinator conservation initiatives in all Massachusetts towns (n = 347) using the terms *pollinat* AND conservation AND [town/city name].* We located relevant groups through community group webpages, social media accounts, newspaper articles, and published conservation plans. We read through each group’s relevant materials (e.g. webpages, published action plans) and extracted the following information: 1) initiative location and name, 2) mission statements or goals, 3) interventions to target pollinators, and 4) specific taxa targeted. We coded goals into three groups according to the following scheme: (i) “ecological and not specific” if a goal to help pollinators was articulated, but no specific outcome or taxa were mentioned, e.g. “support pollinators.” We also coded goals under this group if the ecological goal was actually a management intervention, e.g. “plant native plants.” (ii) We coded goals as “ecological and specific” if a goal was to benefit specific, declining pollinator taxon or group of taxa was mentioned, or if a goal articulated something about restoring species interactions. (iii) And, we coded goals as “social” if goals were human-focused such as public education, improving aesthetics, or enhancing human-nature connection (Figure S1).


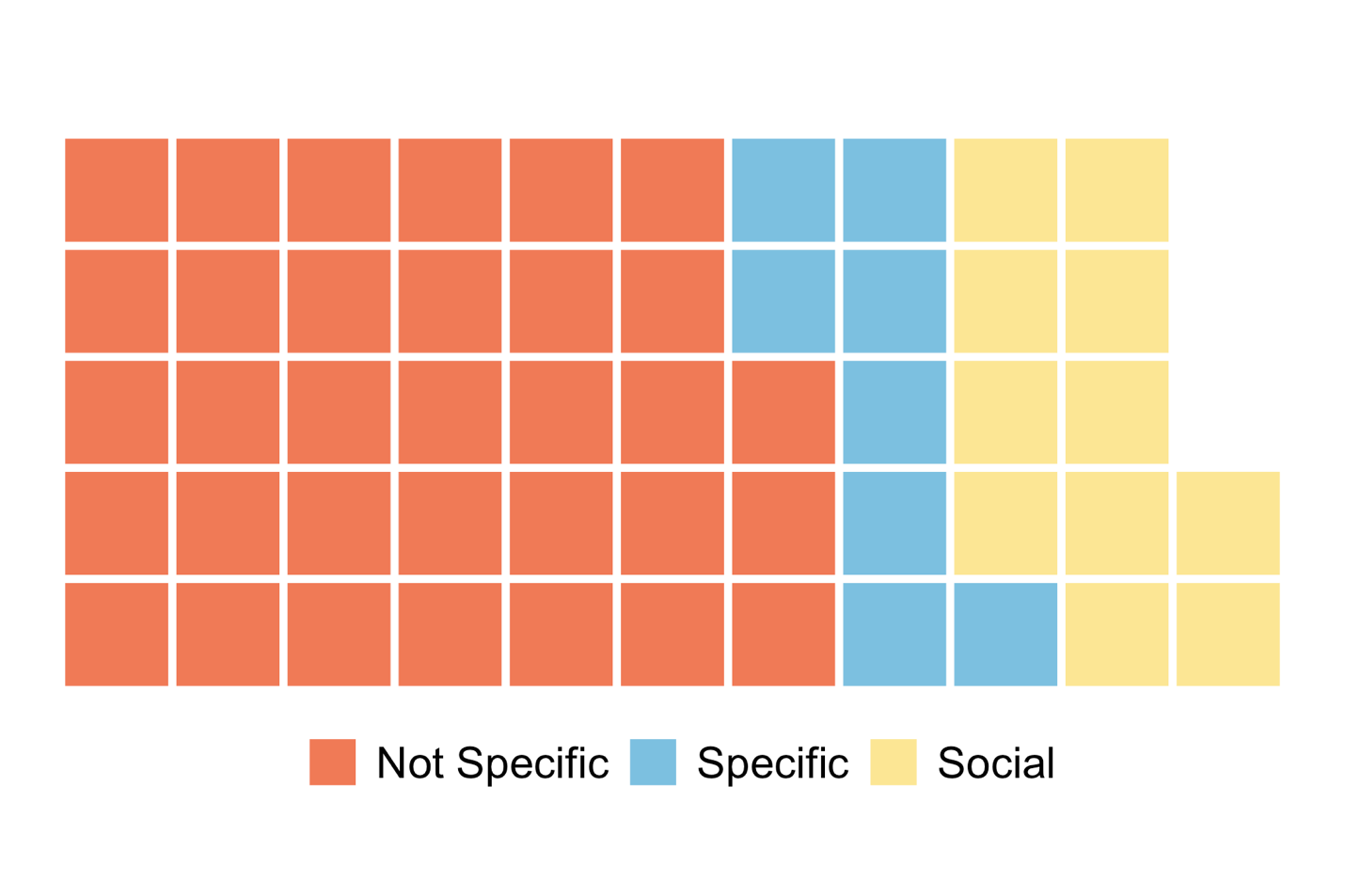


**Figure S1.** Goals set by pollinator gardening initiatives in Massachusetts were classified under ecological goals that were not specific (e.g. “support pollinators”, “promote biodiversity”), ecological goals that were specific (e.g. “restore at-risk bumble bee taxa”), and social goals (e.g. “increase awareness about pollinators”). Each square indicates a single goal (n = 52).
